# Effect of Polar Head Group Modifications on the Tumor Retention of Phospholipid Ether Analogs: Role of the Quaternary Nitrogen

**DOI:** 10.3390/pharmaceutics15010171

**Published:** 2023-01-03

**Authors:** Anatoly N. Pinchuk, Mark A. Rampy, Marc A. Longino, Ben Y. Durkee, Raymond E. Counsell, Jamey P. Weichert

**Affiliations:** 1 Department of Radiology, University of Wisconsin School of Medicine and Public Health, 1111 Highland Ave., WIMR, Madison, WI 53705, USA; 2 Department of Pharmacology, The University of Michigan Medical School, 1150 W. Medical Center Drive, Ann Arbor, MI 48109, USA

**Keywords:** radioiodinated phospholipid ether analogs, alkyl phosphocholines, tumor imaging

## Abstract

We have previously described the remarkable capacity of radioiodinated alkyl phospholipids to be sequestered and retained by a variety of tumors in vivo. We have already established the influence of certain structural parameters of iodinated alkyl phospholipids on tumor avidity, such as stereochemistry at the *sn*-2 carbon of alkylglycerol phosphocholines, *meta*-or *para*-position of iodine in the aromatic ring of phenylalkyl phosphocholines, and the length of the alkyl chain in alkyl phospholipids. In order to determine the additional structural requirements for tumor uptake and retention, three new radioiodinated alkylphospholipid analogs, **2–4**, were synthesized as potential tumor imaging agents. Polar head groups were modified to determine structure-tumor avidity relationships. The trimethylammonio group in **1** was substituted with a hydrogen atom in **2**, an ammonio group in **3** and a tertiary butyl group in **4**. All analogs were separately labeled with iodine-125 or iodine-124 and administered to Walker 256 tumor-bearing rats or human PC-3 tumor-bearing SCID mice, respectively. Tumor uptake was assessed by gamma-camera scintigraphy (for [I-125]-labeled compounds) and high-resolution micro-PET scanning (for [I-124]-labeled compounds). It was found that structural modifications in the polar head group of alkyl phospholipids strongly influenced the tumor uptake and tissue distribution of these compounds in tumor-bearing animals. Phosphoethanolamine analog **3** (NM401) displayed a very slight accumulation in tumor as compared with phosphocholine analog 1 (NM346). Analogs **2** (NM400) and **4** (NM402) lacking the positively charged nitrogen atom failed to display any tumor uptake and localized primarily in the liver. This study provided important insights regarding structural requirements for tumor uptake and retention. Replacement of the quaternary nitrogen in the alkyl phospholipid head group with non-polar substituents resulted in loss of tumor avidity.

## 1. Introduction

**Figure 1:**
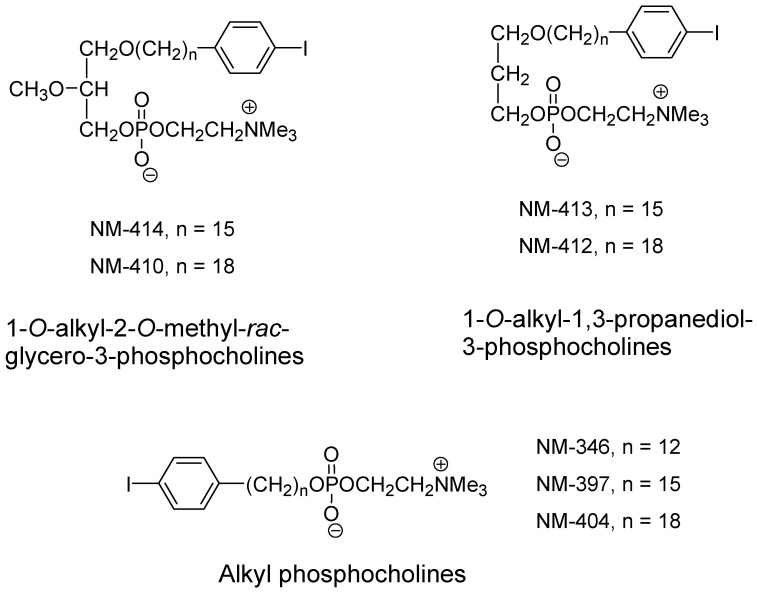
APC Structural classes.

Synthetic phospholipid ether (PLE) and alkyl phosphocholine (APC) analogs are among a class of antitumor agents which do not interact with DNA while inducing apoptosis in cancer cells, minimizing damage to normal cells [1,2,3,4,5,6]. Prior work in our laboratories demonstrated that PLE and APC analogs are selectively sequestered by a variety of animal tumors and human tumor xenografts [3,7,8,9,10,11]. Recently reported SAR effects for several series of phospholipid ether and alkyl phosphocholine analogs demonstrated that: (a) the glycerol backbone is not necessarily required for the tumor avidity, since the hydrophobic portion of the molecule can be simplified to a long-chain alcohol bearing ω-iodophenyl group; (b) the alkyl chain must contain >11 methylene groups; (c) there appears to be no specificity for the position of iodine on the phenyl ring [11]. Previous structure-activity studies identified NM404 (18-(*p*-iodophenyl)octadecyl phosphocholine, Figure 1) as the best tumor imaging agent among nine PLE and APC analogs with tumor-to-background ratios greater than five [11]. When labeled with the appropriate isotope of iodine, NM404 may become a universal tumor-selective theranostic (diagnostic and therapeutic) agent since it displayed significant tumor selectivity and prolonged retention in myriad xenograft and spontaneous primary and metastatic tumors of human and animal origin [3]. It is generally believed that the enhanced uptake of alkylphosphocholines involves lipid membrane rafts found more highly concentrated in tumor cell membranes [3,5,6]. In vitro studies conducted with ^125^I-NM404 in PC-3 prostate cancer cells pretreated with Filipin III, an agent that sequesters cholesterol and thus disrupts membrane lipid raft integrity, resulted in a 40% reduction in ^125^I-NM404 uptake compared with untreated PC-3 cells, thus supporting the role of lipid membrane rafts in tumor accumulation of APC analogs [3]. Furthermore, a 60% reduction in the uptake of optically active BODIPY-NM404 was observed by confocal microscopy in A549 NSCLC tumor cells pretreated with methyl-β-cyclodextrin, a selective disrupter of membrane lipid raft activity [3]. Therefore, entry of NM404 into cancer cells is likely facilitated by membrane lipid rafts overexpressed in tumors resulting in the observed selective sequestration and retention of APC analogs in both primary and metastatic lesions regardless of anatomic location, even those in brain and lymph nodes.

Initial studies aimed at determining the influence of molecular structure on the tumor retention of PLE and APC analogs indicated that for glycerol-derived PLE analogs, the stereochemistry at the *sn*-2 position of glycerol was not a factor in the tumor uptake and retention: *R*- and *S*-enantiomers showed no difference in tumor uptake [10]. Moreover, alkyl chain length appears to be a key determinant in tumor retention of these compounds as it alters the hydrophobic properties of these analogs. As reported, decreasing the chain length from C12 to C7 minimized tumor uptake, however, chain length increases led to enhanced tumor sequestration with concomitant delay of plasma clearance [11].

Synthetic PLE and APC analogs with the following modifications in the polar part of the phospholipid molecule have been described in the literature: heterocyclic analogs [12,13,14,15,16,17,18,19], nucleoside conjugates [20], 2-(2-trimethylammonioethoxy)ethyl and congeneric oligo(ethyleneoxy)ethyl analogs [21], 2-(trimethylarsonio)ethyl [22], 2-(trimethylphosphonio)ethyl [22] and (dimethylsulfonio)ethyl analogs [23], analogs with carbon-phosphorus bonds [24,25], analogs with an increased number of methylene groups between quaternary nitrogen and phosphate group [12,18,19,26,27], as well as ethanolamine [28,29], serine [28,29,30,31,32], threonine [32] and inositol [33,34] analogs. A series of glycosylated antitumor ether lipids (GAELs) which kill cancer cells by an apoptosis-independent mechanism has recently been reported [35,36]. Most of these compounds were synthesized for structure-activity studies in an attempt to improve antineoplastic properties and other biological activities of PLE and APC analogs but no information was provided relating to their biodistribution properties. Furthermore, none of these compounds were radiolabeled and all were tested at much higher mass doses than our radioiodinated PLE and APC analogs.

**Figure 2:**
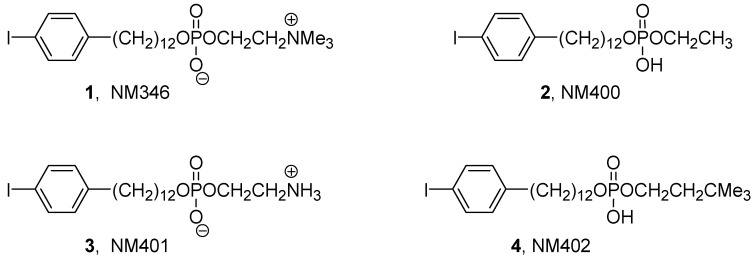
Structures of polar head group modified alkyl phospholipids.

Further analysis of the structural requirements for selective accumulation of PLE and APC analogs in tumor cells led to the present study to address the role of the quaternary nitrogen in the polar head group of phospholipid molecule in the tumor uptake. Here, we report the synthesis, tissue distribution, and tumor imaging studies of three radioiodinated alkyl phospholipid analogs **2**, **3** and **4** (Figure 2). Their polar head groups have variations in steric bulk and polarity. In these compounds, the choline part of the molecule is substituted with the moieties of ethanol in **2**, ethanolamine in **3** and 3,3-dimethyl-1-butanol in **4**. Due to the ease of the synthesis, and availability of 12-(*p*-iodophenyl)dodecanol **5** in our lab, we have decided to limit the alkyl chain length to 12 methylene groups in this comparison.

## 2. Materials and Methods

### 2.1. Chemistry

All chemicals and solvents were acquired from Aldrich Chemical Co. (Milwaukee, WI, USA). Silica Gel 60 F_254_ plates (MilliporeSigma, Burlington, MA, USA) were employed for analytical thin-layer chromatography. Visualization of developed plates was achieved by illumination under 254 nm UV light and/or by charring after immersion in cerium molybdate stain followed by heating on a hot plate. For flash chromatography, silica gel 32–63 µm from Fisher Scientific (Hanover Park, IL, USA) was used. NMR data were collected on Unity Inova 400 and 500 spectrometers. Chemical shifts are reported in parts per million (ppm) relative to tetramethylsilane (TMS), and spin multiplicities are given as s (singlet), t (triplet), dt (doublet of triplets), q (quartet), m (complex multiplet). Elemental analyses of the compounds agreed to within ±0.4% of the calculated value. High-resolution mass spectra were obtained on an IonSpec 7 Tesla HiResMALDI FT-Mass Spectrometer at the Analytical Instrumentation Center of University of Wisconsin School of Pharmacy.

**12-(*p*-Iodophenyl)dodecyl phosphocholine (1)** was synthesized as described earlier by Rampy et al. [9].

**12-(*p*-Iodophenyl)dodecyl-*H*-phosphonate (6).** To a solution of imidazole (1 g, 14.7 mmol) in methylene chloride (13 mL) at 0 °C was added phosphorus trichloride (0.28 mL, 3.2 mmol) in methylene chloride (3 mL) for 10 min followed by triethylamine (1.2 mL, 8.3 mmol). Reaction mixture was stirred for 10 min at 0 °C, then cooled to −15 °C and 12-(*p*-iodophenyl)dodecanol **5** [9] (420 mg, 1.08 mmol) in methylene chloride (15 mL) was added slowly over 30 min. After stirring for additional 30 min, the reaction mixture was quenched by 1M triethylammonium bicarbonate buffer solution (3 mL). After 10 min, the mixture was diluted with MeOH-H_2_O mixture (1:1, 70 mL). Organic layer was separated and washed with water. Aqueous layer was extracted with chloroform (2 × 35 mL) and extracts were washed with water. Extracts were dried over Na_2_SO_4_ and evaporated. The residue was purified by silica gel chromatography in CHCl_3_-MeOH-Et_3_N (95:5:1) to afford the product, 12-(*p*-iodophenyl)dodecyl-*H*-phosphonate triethylammonium salt. This compound was dissolved in CHCl_3_ (20 mL) and MeOH (20 mL) and washed with 0.05 N HCl (18 mL). Chloroform extract was dried over Na_2_SO_4_ and evaporated to give 12-(*p*-iodophenyl)dodecyl-*H*-phosphonate, 340 mg (70%); ^1^H NMR (400 MHz, CDCl_3_–CD_3_OD 1:1): 7.58 and 6.95 (two dt*, J* = 8.4, 1.9 Hz, 2H each, IC_6_H_4_), 6.73 (d, *J* = 622 Hz, P-H), 3.83 (q, *J* = 7.0 Hz, 2H, CH_2_OP), 2.56 (t, *J* = 7.6 Hz, 2H, ArC*H_2_*), 1.66–1.54 (m, 4H, ArCH_2_C*H_2_* and C*H_2_*CH_2_OP), 1.40–1.24 (m, 16H, (CH_2_)_8_); ^31^P NMR (161.8 MHz, CDCl_3_–CD_3_OD 1:1): 5.90. Anal. (C_18_H_30_IO_3_P) C, H, N.

**12-(*p*-Iodophenyl)dodecyl phosphoethanolamine (3).** To a solution of dried 12-(*p*-iodophenyl)dodecyl-*H*-phosphonate **6** (95 mg, 0.21 mmol) and *N*-BOC-ethanolamine (0.1 mL, 0.65 mmol) in pyridine (2.5 mL) was added NPCl (116 mg, 0.63 mmol). The reaction mixture was stirred at room temperature for 1 h and a solution of iodine (100 mg, 0.39 mmol) in pyridine—THF—water (5:5:1, 1.1 mL) was added. The reaction mixture was stirred for 10 min, quenched with Na_2_S_2_O_3_ solution, and mixed with CHCl_3_ (20 mL), MeOH (20 mL) and water (15 mL). The chloroform layer was separated, and the aqueous layer was extracted again with CHCl_3_ (2 × 20 mL). Extracts were combined, dried over Na_2_SO_4_ and evaporated. The residue was dissolved in methylene chloride (1 mL) and cooled to 0 °C before trifluoroacetic acid (1 mL) and 70% perchloric acid (1 mL) were added. The reaction mixture was stirred for 20 min and quenched by careful addition of saturated aqueous NaHCO_3_ solution (20 mL). After gas evolution had ceased, the reaction mixture was poured into the mixture of CHCl_3_ (20 mL) and MeOH (20 mL). The reaction mixture was mixed with CHCl_3_ (20 mL), MeOH (20 mL) and water (15 mL), chloroform layer was separated, and aqueous layer was extracted again with CHCl_3_ (2 × 20 mL). After drying the organic phase over Na_2_SO_4_ and evaporation of solvents, the residue was purified by silica gel chromatography in CHCl_3_–MeOH–H_2_O (65:25:2) to yield the product after precipitating with acetone, as a white powder (70 mg, 65%); ^1^H NMR (500 MHz, CDCl_3_–CD_3_OD 2:1): 7.58 and 6.94 (two dt*, J* = 8.3, 1.9 Hz, 2H each, IC_6_H_4_), 4.07–4.02 (m, 2H, POC*H_2_*CH_2_N), 3.87 (q, *J* = 6.6 Hz, 2H, C*H_2_*OPOCH_2_CH_2_N), 3.13–3.09 (m, 2H, CH_2_N), 2.55 (t, *J* = 7.7 Hz, 2H, ArC*H_2_*), 1.66–1.54 (m, 4H, ArCH_2_C*H_2_* and C*H_2_*CH_2_O), 1.40–1.24 (m, 16H, (CH_2_)_8_). HRMS: calculated for C_20_H_36_INO_4_P (M + H^+^) 512.1421, found 512.1396. Anal. (C_20_H_35_IO_4_P) C, H, N.

**12-(*p*-Iodophenyl)dodecyl phospho-[3,3-dimethyl-1-butyl] ester (4).** To a solution of dried 12-(*p*-iodophenyl)dodecyl-*H*-phosphonate **6** (110 mg, 0.24 mmol) and 3,3-dimethyl-1-butanol (0.072 mL, 0.6 mmol) in pyridine (2 mL) was added NPCl (110 mg, 0.6 mmol). The reaction mixture was stirred for 40 min at room temperature and a solution of iodine (100 mg, 0.39 mmol) in pyridine—THF—water (5:5:1, 1.1 mL) was added. After 10 min, the reaction was quenched with Na_2_S_2_O_3_ solution and transferred into separation funnel with CHCl_3_ (20 mL), MeOH (20 mL) and H_2_O (15 mL). The chloroform layer was separated and aqueous phase was extracted with additional CHCl_3_ (2 × 20 mL). Extracts were combined, dried over Na_2_SO_4_ and evaporated. The residue was purified by silica gel chromatography in CHCl_3_–MeOH—25% aqueous ammonium hydroxide (9:1:0.05) to give 104 mg (76%) of the product as the ammonium salt; ^1^H NMR (500 MHz, CDCl_3_–CD_3_OD 2:1): 7.58 and 6.94 (two dt*, J* = 8.1, 1.8 Hz, 2H each, IC_6_H_4_), 3.94–3.88 (m, 2H, POC*H_2_*CH_2_Cme_3_), 3.84 (q, *J* = 6.4 Hz, 2H, C*H_2_*OPOCH_2_CH_2_Cme_3_), 2.55 (t, *J* = 7.7 Hz, 2H, ArC*H_2_*), 1.66–1.54 (m, 4H, ArCH_2_C*H_2_* and C*H_2_*CH_2_O), 1.59 (t, *J* = 7.8 Hz, 2H, C*H_2_*Cme_3_), 1.42–1.24 (m, 16H, (CH_2_)_8_), 0.94 (s, 9H, C(CH_3_)_3_). HRMS: calculated for C_24_H_42_InaO_4_P (M + Na^+^) 575.1758, found 575.1779. Anal. (C_24_H_45_INO_4_P, ammonium salt) C, H, N.

**12-(*p*-Iodophenyl)dodecyl phospho-ethyl ester (2).** This compound was synthesized utilizing the same procedure described for **4** from 12-(*p*-iodophenyl)dodecyl-*H*-phosphonate **6** (82 mg, 0.18 mmol), absolute ethanol (0.03 mL, 0.53 mmol) and NPCl (97 mg, 0.53 mmol) in pyridine (2 mL) to give 71 mg (77%) of the product as the ammonium salt; ^1^H NMR (500 MHz, CDCl_3_–CD_3_OD 2:1): 7.58 and 6.94 (two dt*, J* = 8.1, 1.8 Hz, 2H each, IC_6_H_4_), 3.92 (quintet, *J* = 7.0 Hz, 2H, POC*H_2_*CH_3_), 3.84 (q, *J* = 6.4 Hz, 2H, C*H_2_*OPOCH_2_CH_3_), 2.55 (t, *J* = 7.7 Hz, 2H, ArC*H_2_*), 1.65–1.55 (m, 4H, ArCH_2_C*H_2_* and C*H_2_*CH_2_O), 1.40–1.24 (m, 19H, (CH_2_)_8_ and POCH_2_C*H_3_*). HRMS: calculated for C_20_H_34_InaO_4_P (M + Na^+^) 519.1132, found 519.1141. Anal. (C_20_H_37_INO_4_P, ammonium salt) C, H, N.

### 2.2. Radioiodination of Phospholipid Analogs

*Labeling with Iodine-125.* Radioiodination of compounds **1–4** with iodine-125 was accomplished as previously reported [37]. Radiochemical purity was established by radio-TLC with unlabeled material serving as a reference standard. Specific activity for compound **1–4** ranged from 0.5 to 3.0 Ci/mmol.

*Labeling with Iodine-124.* Phospholipid analogs **1–4** were radiolabeled with iodine-124 via a modified isotope exchange reaction utilizing ammonium sulfate as the exchange medium [38]. Briefly, the phospholipid analog (30 µg in 30 µL of ethanol) was added to a glass vial containing ammonium sulfate (10 mg) dissolved in 20 µL of deionized water. Following addition of sodium iodide-124 (up to 2 mCi in <50 µL 0.1N NaOH, IBA Molecular, Reston, VA, USA), the resulting mixture was diluted with 200 µL of ethanol and a tandem coconut charcoal/glass wool trap apparatus inserted via 18-gauge needle into the reaction vial. The reaction vial was heated at 145 °C for 45 min, 50 mL of air injected into the reaction vial, and then subsequently heated at 155 °C for an additional 30 min. After cooling, ethanol (300 µL) was added to the resulting dry residue and the reaction vial vortexed briefly to aid extraction of the PLE analog. The suspension was filtered and solvent evaporated. The remaining residue was dissolved in 70 µL of ethanol (NM346, NM401) or alternatively in chloroform/methanol/water (65:10:1) (NM400, NM402) prior to HPLC purification.

*HPLC Purification and Formulation of Radioiodinated Phospholipids.* Preparative HPLC purification was performed on a Gilson System HPLC using a silica gel cartridge column (33 × 4.6 mm, 3 μm, Perkin Elmer) with a guard column (Hypersil Silica, 10 × 4.6 mm, 5 μm, Supelco, Bellefonte, PA, USA) and eluted (1 mL/min) with either isopropanol/hexanes/water (52:40:8) for NM346 and 401 or chloroform/methanol/water (65:10:1) for NM400 and 402. Radioactivity and UV (230 and 254 nm) were used for detection and pure fractions were collected for each radiolabeled compound. Radiochemical purity exceeded 98% in all cases and radiochemical yields ranged from 20% to 70%. Following solvent removal in vacuum, the remaining pure radioiodinated compound was solubilized in water using Tween-20 (2%) as a surfactant. The final solution was filtered (0.22 µm) prior to injection. For more details on formulation, see Ref. [11].

### 2.3. Biology

*Cell Lines, Culture Conditions and Animals.* All information on these subjects is provided in Ref. [11]. PC-3 prostate tumor cells were obtained from ATCC (American Type Culture Collection). All procedures using animals conformed strictly to the guidelines set forth by the animal care units of each respective institution, which reviewed and approved the experimental protocol.

**Figure 3:**
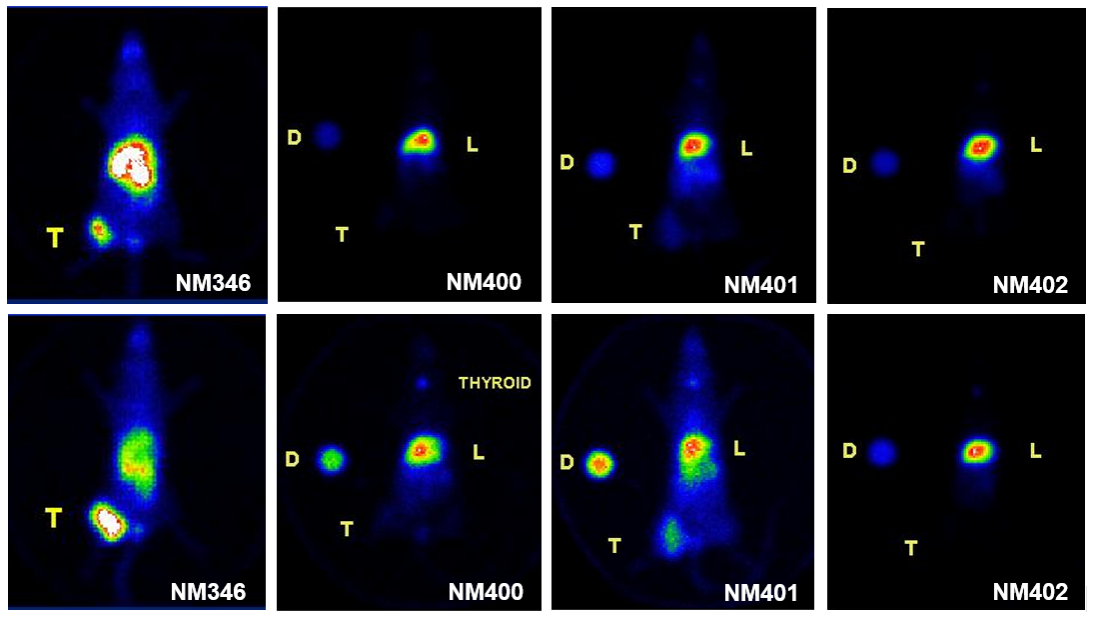
Representative gamma camera planer scans of rats bearing the Walker-256 carcinosarcoma at 24 h (**top row**) and 120 h (**bottom row**) following administration of iodine-125 labeled alkyl phospholipid polar head group analogs **1** (NM346), **2** (NM400), **3** (NM401) and **4** (NM402). L = liver and abdominal activity, D = dose standard, T = tumor.

*Gamma Camera Scintigraphy and Tissue Distribution.* Walker tumor-bearing rats (n = 3 per compound) received formulated alkyl phospholipid analogs labeled with iodine-125 (30–50 μCi per animal in 0.5 mL of 2% Tween saline) administered intravenously via tail vein. Dose standards (1, 2, 5, 10% of injected dose) were prepared for use during whole body gamma scanning of the animal. Dose standards consisted of 10 mL physiological saline and radiolabeled phospholipid analog in 20 mL polyethylene (HDPE) scintillation vials. Standards were placed on the camera with the animal during the imaging protocol. Sedation of tumor-bearing animals was achieved by i.m. administration of ketamine (87 mg/kg) and xylazine (13 mg/kg). Scanning was performed with a Siemens LEM Mobile camera fitted with a high sensitivity-low energy collimator able to detect the low-energy (35 keV) gamma rays emitted by iodine-125. Image acquisition and data storage was achieved with a Siemens MicroDELTA/ Micro VAX computer system. Images (20 min acquisition) were obtained at 24 and 120 h after administration of the radiolabeled agents (Figure 3). Following the 120 h scan, each animal was sacrificed for subsequent and tissue distribution analysis as described in Ref. [11].

**Figure 4:**
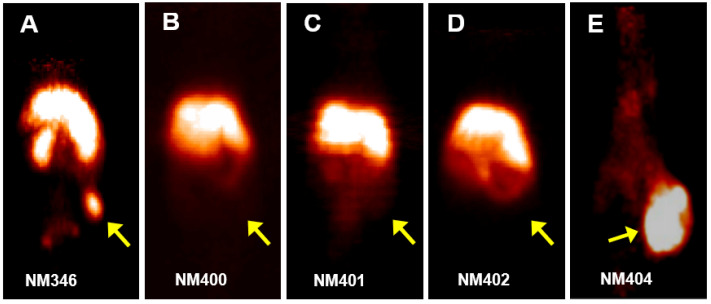
**The** 3D-microPET projection images obtained in human PC-3 prostate tumor-bearing mice 24 h after i.v. injection of iodine-124 labeled alkyl phospholipid polar head group analogs NM346 (**A**), NM400 (**B**), NM401 (**C**) and NM402 (**D**). Although NM404 is not a C12 analog, its image (**E**) is included for comparison. Tumor indicated by the arrow. Compared to NM346 which affords significant liver and gut activity as well as some tumor uptake, analogs NM400, NM401 and NM402 display similar liver and gut activity but no tumor uptake.

*MicroPET Scanning.* The iodine-124 labeled alkyl phospholipid analogs (60–80 µCi in 200 µL) were injected intravenously (lateral tail vein) into human PC-3 tumor-bearing SCID mice (n = 2 for each compound). Tumor-bearing mice were scanned 24 h after injection of the respective iodine-124 labeled analog on a Concorde Microsystems R4 microPET scanner (CTI/Siemens, Knoxville, TN, USA) utilizing a 20 min acquisition time and filtered back projection image reconstruction. Three-dimensional projection images (Figure 4) were used for comparison in order to show all radioactivity in an animal simultaneously.

## 3. Results and Discussion

**Scheme 1:**
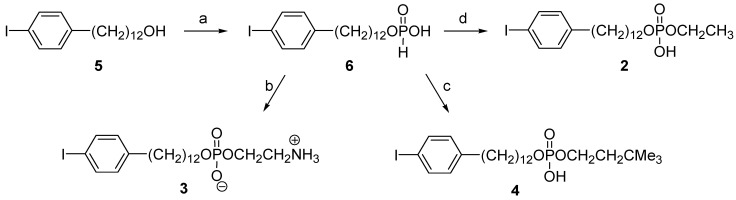
Reagents, solvents and conditions: (a) (i) PCl_3_, Im, Et_3_N, CH_2_Cl_2_, -15 °C, 30 min, (ii) aqueous TEAB buffer, 70%; (b) (i) *N*-BOC-ethanolamine, NPCl, Py, room temp., 1 h, (ii) I_2_, Py-THF-H_2_O, room temp., 10 min, (iii) CF_3_CO_2_H-HClO_4_, CH_2_Cl_2_, 0 °C, 20 min, 65%; (c) (i) 3,3-dimethyl-1-butanol, NPCl, Py, room temp, 40 min, (ii) I_2_, Py-THF-H_2_O, room temp., 10 min, 76%; (d) ethanol, NPCl, Py, room temp, 40 min, (ii) I_2_, Py-THF-H_2_O, room temp., 10 min, 77%.

Phospholipids **2**, **3** and **4** were synthesized using the *H*-phosphonate method [39,40] (Scheme 1). This method was chosen because it allows using a common intermediate, *H*-phosphonate monoester **6**, that does not require any protecting group on the phosphorus center. Phosphitylation of 12-(*p*-iodophenyl)dodecanol **5 [9]** with PCl_3_/imidazole gave 12-(*p*-iodophenyl)dodecyl-*H*-phosphonate **6**. In the next step, *H*-phosphonate **6** was coupled with the second alcohol component (*N*-BOC-ethanolamine, 3,3-dimethyl-1-butanol or ethanol) in the presence of an activating agent NPCl, (2-chloro-5,5-dimethyl-1,3,2-dioxaphosphorinane 2-oxide). Without isolation, the intermediate *H*-phosphonate diester was oxidized to the phosphodiester by iodine in pyridine–THF–water. This one-pot procedure afforded compounds **2** and **4** which were isolated after chromatography as ammonium salts. In the synthesis of ethanolamine analog **3**, the *N*-BOC phosphodiester intermediate was subjected to deprotection to provide **3.**

To evaluate the capacity of new alkyl phospholipid head group analogs to accumulate in and visualize tumors, compounds **1–4** were labeled separately with two different isotopes of radioiodine and evaluated in two different tumor models. In the first series of experiments, compounds were radiolabeled with iodine-125 via an isotope exchange in pivalic acid [37]. Tumor imaging was performed in Walker-256 tumor-bearing rats at 24 and 120 h following i.v. administration of each radioiodinated analog (Figure 3). NM346 (**1**), the alkyl phosphocholine analog, accumulated in the Walker-256 tumor, permitting its visualization. Although an acceptable image of the tumor was obtained at 24 h, additional radioactivity was still observed in the abdominal area and bladder. By 120 h the abdominal activity had essentially cleared leaving most of the remaining radioactivity in the tumor. In contrast, analog **2** (NM400, the phosphoethanol derivative) and **4** (NM402, the carbon isostere of choline) concentrated primarily in the abdominal region (liver and gastrointestinal tract) with no visible uptake in the tumor at either time point. Compound **3** (NM401, the ethanolamine derivative) displayed a slight degree of accumulation in the tumor after 24 h with only slight enhancement by 120 h but to a much lesser extent than NM346.

At the end of the 120 h gamma camera scanning protocol, the animals were euthanized and subjected to tissue distribution analyses. Biodistribution results are summarized in Table 1. NM346 (**1**) had the highest levels of radioactivity in the blood, duodenum, lung and tumor. Tumor levels of radioactivity after administration of NM346 were 2.04 ± 0.18% ID/g as compared with NM401 (**3**) which had a 0.36 ± 0.08% ID/g in the tumor. NM400 and NM401 had very low tumor radioactivity levels (<0.1% ID/g). Although NM402 did not accumulate in the tumor, it displayed the highest levels radioactivity in the liver (3.3 ± 0.5% ID/g, 25.3 ± 2.5% dose/organ). NM400 (1.3 ± 0.07% ID/g, 15.4 ± 1.0% dose/organ) and NM402 (1.14 ± 0.17% ID/g, 10.7 ± 0.8% dose/organ) had much lower levels of activity in the liver. NM346 possessed liver levels of radioactivity (0.84 ± 0.12% ID/g, 11.79 ± 1.5% dose/organ) similar to those of NM401.

In the second series of experiments, phospholipids **1–4** were labeled with iodine-124 (a positron emitting isotope) via an isotope exchange reaction using ammonium sulfate as the exchange medium [38]. Iodine-124 labeled analogs **1–4** were administered to human PC-3 tumor-bearing SCID mice and microPET images were obtained. Figure 4 shows microPET scans of the animals obtained 24 h after i.v. administration of radioiodinated polar head group analogs **1–4**. Alkyl phosphocholine analog NM346 (**1**) accumulated to a modest extent in the tumor and afforded its visualization. As in the previous set of experiments with iodine-125 labeled compounds, significant activity in liver and intestinal tract was observed with [I-124]-NM346. Iodine-124 labeled analogs without nitrogen in the polar head group, NM400 and NM402 localized almost exclusively in the abdominal area of the animal and failed to accumulate in the tumor at the 24 h time point.

MicroPET imaging studies with iodine-124 labeled compounds in the PC3 prostate tumor xenograft mouse model provided results similar to the gamma camera scintigraphy with iodine-125 labeled compounds in the rat Walker-256 carcinosarcoma model.

## 4. Rationale

The gamma emitter, ^125^I (35 keV) was the most suitable probe for the preliminary planar scintigraphy imaging studies of the labeled alkyl phospholipid analogs with the Walker 256 tumor model. The subsequent 3-D imaging by micro-PET with ^124^I-labeled analogs in the PC-3 tumor-bearing mice provided volume imaging of the animal to give a better picture of the compound distribution.

The Walker 256 carcinosarcoma model (a rat mammary tumor line) was routinely used to determine biodistribution of ^125^I-labeled APC analogs. The Walker 256 rat model also permitted 2-D planar scintigraphic imaging with the same labeled analogs given the inherent resolution limitations of the technique. As research shifted to 3-D volume imaging by microPET with the corresponding ^124^I-labeled APC compounds, the PC-3 SCID mouse model was more suited to the microPET device, allowing for exquisite depiction of in vivo biodistribution of the labeled compounds.

A specific rationale was used in the design of each of the polar head group analogs. In NM400 molecule, the hydrogen atom is substituted for the trimethylammonio group present in NM346. As the data shows, NM400 had cleared from a majority of the tissues with significant uptake in the duodenum, kidney, liver, plasma and thyroid. Most of this activity was associated with the liver. Previous work by Bishop and coworkers [41] demonstrated that ether lipid analogs such as hexadecylphosphocholine can be broken down in a fashion consistent with the action of phospholipase D. Some years ago, we have investigated the breakdown of alkylphosphocholines such as NM324 (*meta*-iodophenyl isomer of NM346) by phospholipase D [42]. In the presence of trace quantities of ethanol, a phosphoethanol derivative (similar to NM400) could be generated. In fact, nothing concerning the biological disposition of exogenously administered phosphoethanol derivatives has been reported to date.

The disposition of NM401, the ethanolamine derivative, was evaluated in tumor-bearing animals for several reasons. Since it is a zwitterion, the charge distribution in the polar head group would be similar to the tumor avid NM346 although the steric environment of nitrogen atom in NM401 is different. As a result, the overall size and hydration of the polar head group of NM401 would be expected to be quite different from NM346. In addition, phosphatidylethanolamine is the second most common phospholipid component of mammalian cell membranes and has been reported to be elevated in certain types of cancer [43]. Of the four analogs tested, NM346 had the greatest tumor avidity. NM401 displayed a very slight accumulation in the tumor, however this uptake of radioactivity did not afford an adequate tumor-to-nontarget ratio.

The third compound, NM402, was designed to evaluate the importance of the positively charged quaternary nitrogen atom in the phosphocholine head group. This was accomplished by replacing the positively charged quaternary nitrogen with a tetrasubstituted carbon atom. 3,3-Dimethyl-1-butanol can be viewed as a choline isostere in which positively charged trimethylammonium group is substituted with a neutral tertiary butyl group. This substitution results in a slight change in molecular weight of the compound and produces a phospholipid molecule with a negative charge on the phosphate. NM402 did not demonstrate any tumor avidity and instead displayed extensive accumulation (3.3% ID/g) in the liver as late as 120 h after administration (Table 1).

As mentioned previously, we have shown bulk tolerance in the aromatic ring of these radioiodinated phospholipid analogs illustrated by the fact that replacement of the aromatic iodine even with large near-infrared imaging moieties and metal chelates does not impede tumor uptake of these APC analogs which have stimulated use of these agents as real time surgical tumor margin illuminators and as cancer theranostics and immune stimulators, respectively [3,44,45,46,47].

## 5. Conclusions

Additional understanding of the role of the APC quaternary nitrogen, its steric environment and the significance of the polar head charge on uptake and retention of these analogs has been obtained. The presence of the quaternary nitrogen in the polar head group is critical to the tumor avidity of the APC molecule as evidenced by the inability of the negatively charged analogs NM400 and NM402 to accumulate in tumor cells. This is crucial information in the ongoing development of the alkyl phosphocholine platform for multimodal tumor imaging and therapy.

## Figures and Tables

**Table 1:** Tissue distribution of ^125^I-labeled APC analogs in tumor-bearing rats 120 h after i.v. injection *^a^*.

Tissue	1 (NM346)	2 (NM400)	3 (NM401)	4 (NM402)
Adrenal	-----	0.04 ± 0.01	0.18 ± 0.02	0.05 ± 0.00
Blood	0.22 ± 0.03	0.07 ± 0.00	0.19 ± 0.05	0.04 ± 0.01
Bone Marrow	-----	0.05 ± 0.01	0.13 ± 0.01	0.03 ± 0.01
Duodenum	0.80 ± 0.06	0.13 ± 0.01	0.32 ± 0.06	0.40 ± 0.02
Fat	-----	0.03 ± 0.02	0.11 ± 0.03	0.02 ± 0.00
Heart	-----	0.03 ± 0.00	0.11 ± 0.02	0.02 ± 0.00
Kidney	-----	0.82 ± 0.02	1.32 ± 0.23	0.31 ± 0.03
Liver	0.84 ± 0.12	1.30 ± 0.07	1.14 ± 0.17	3.30 ± 0.48
Lung	0.68 ± 0.11	0.07 ± 0.01	0.26 ± 0.05	0.04 ± 0.00
Muscle	-----	0.02 ± 0.00	0.06 ± 0.02	0.02 ± 0.00
Ovary	-----	0.06 ± 0.00	0.17 ± 0.03	0.04 ± 0.01
Plasma	-----	0.10 ± 0.00	0.30 ± 0.09	0.06 ± 0.01
Spleen	-----	0.04 ± 0.00	0.16 ± 0.03	0.02 ± 0.00
Thyroid	13.95 ± 0.80	49.30 ± 21.59	19.21 ± 3.65	28.88 ± 3.31
Tumor	2.04 ± 0.18	0.07 ± 0.03	0.36 ± 0.08	0.04 ± 0.01
Tumor/Blood	9.27	1.00	1.89	1.00
Tumor/Kidney	-----	0.09	0.27	0.13
Tumor/Liver	2.43	0.05	0.32	0.01

*^a^* Results are expressed as mean % administered dose per gram ± s.e.m. (n = 3).

## Data Availability

Data are contained within the article and Supplementary Materials.

## Supplementary Materials

The following supporting information can be downloaded at: https://www.mdpi.com/article/10.3390/pharmaceutics15010171/s1, ^1^H-NMR spectra of compounds **6**, **2**, **3** and **4**.
