# Supplementary material for: Effect of Polar Head Group Modifications on the Tumor Retention of Phospholipid Ether Analogs: Role of the Quaternary Nitrogen
Source: Pharmaceutics. 2023 Jan 3;15(1):171. doi: 10.3390/pharmaceutics15010171 (PMC9865954; doi:10.3390/pharmaceutics15010171)
Supplement: Supplementary file 1 [file pharmaceutics-15-00171-s001.zip › pharmaceutics-2051391-supplementary.pdf]

Supporting Information

Effect of Polar Head Group Modifications on the Tumor Retention of Phospholipid Ether Analogs: Role of the Quaternary Nitrogen

Anatoly N. Pinchuk [1,*], Mark A. Rampy [2,†], Marc A. Longino [1], Ben Y. Durkee [1], Raymond E. Counsell [2] and Jamey P. Weichert [1]

1. Department of Radiology, University of Wisconsin School of Medicine and Public Health, 1111 Highland Ave., WIMR, Madison, WI 53705, USA
2. Department of Pharmacology, The University of Michigan Medical School, 1150 W. Medical Center Drive, Ann Arbor, MI 48109, USA

* Correspondence: apinchuk@uwhealth.org

† Current address: Sequella Inc., 9610 Medical Center Drive, Suite 200, Rockville, MD 20850, USA.

Table of Contents:

^{1}H-NMR spectra of compounds **6**, **2**, **3** and **4**……………………………………S2 – S5

S2

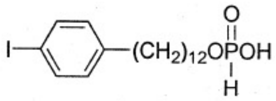

6

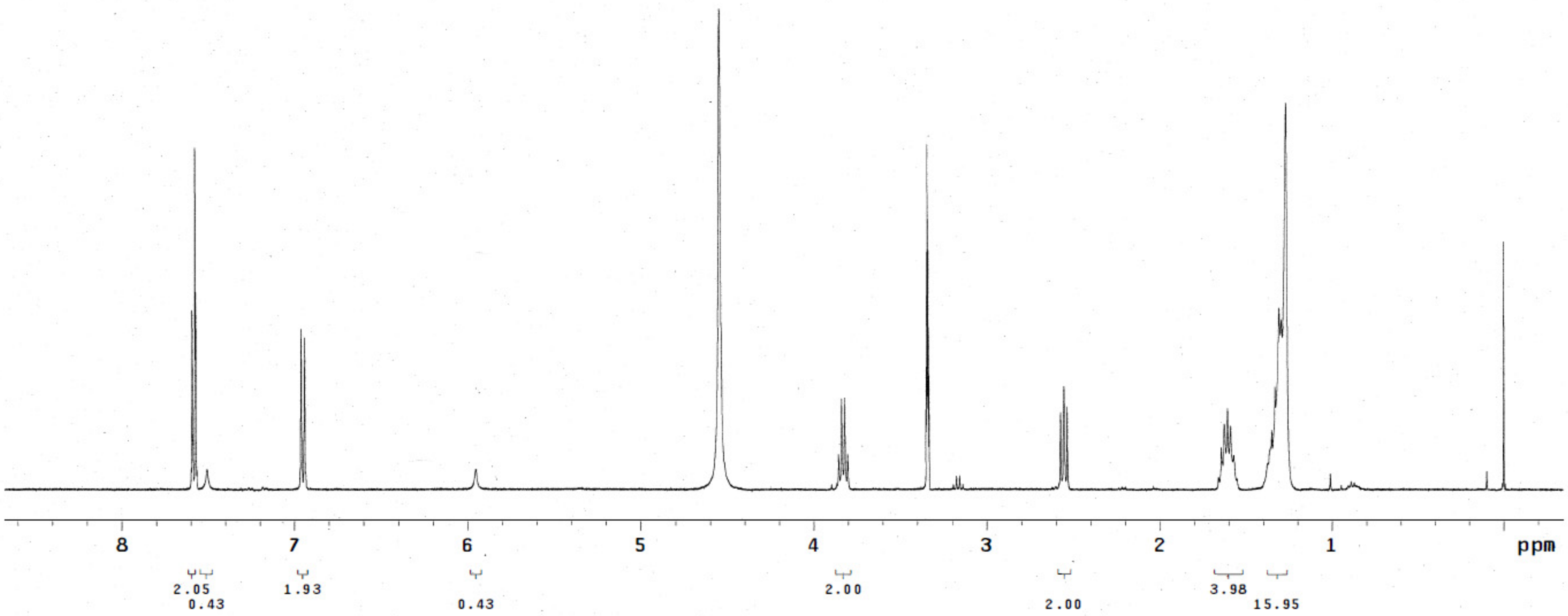

S3

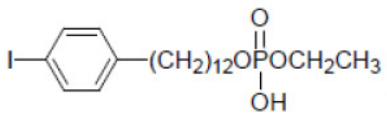
I
(CH2)12OPOCH2CH3
O
OH

2

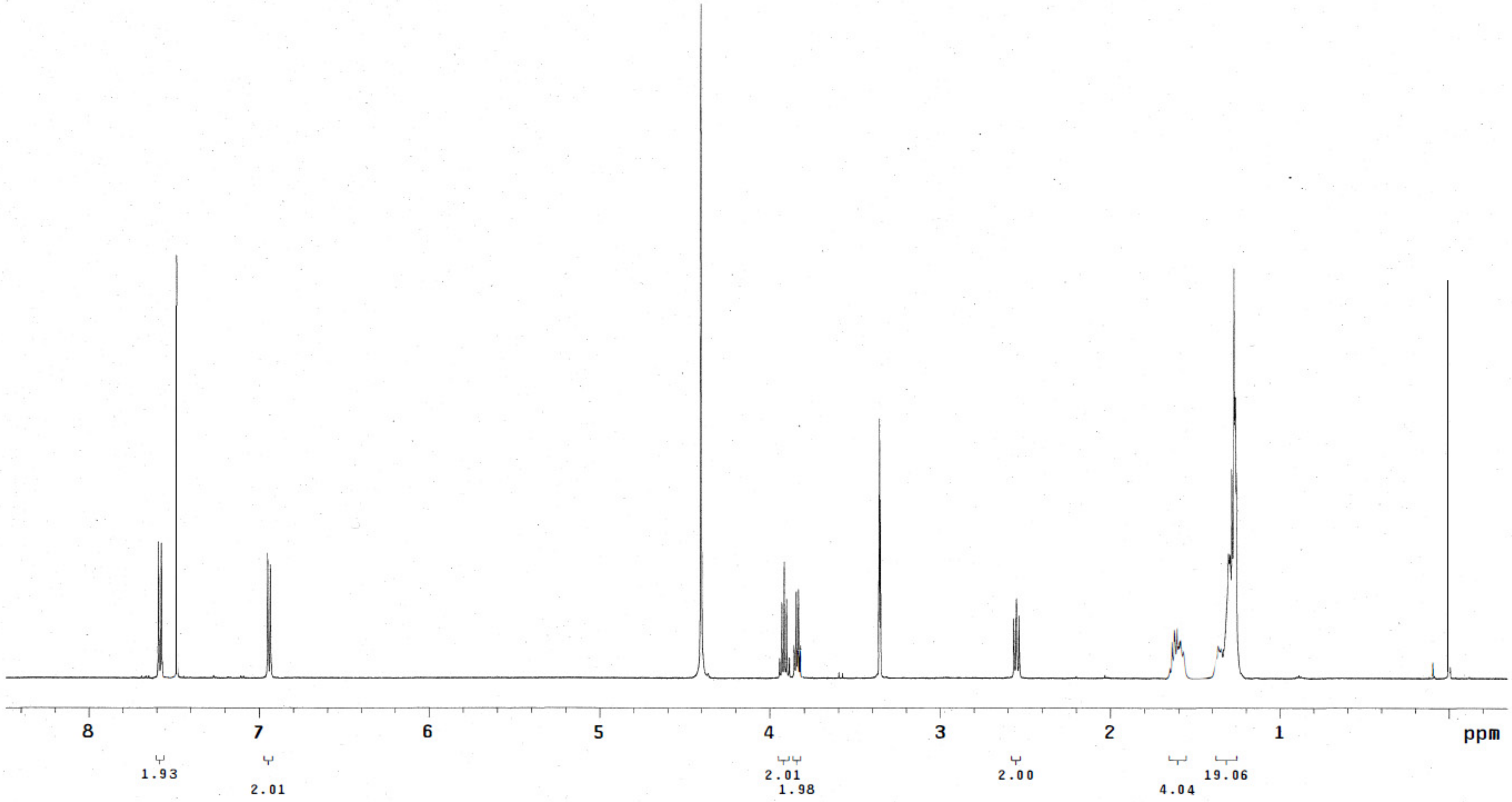
8
7
6
5
4
3
2
1
ppm
1.93
2.01
2.01
1.98
2.00
4.04
19.06

S4

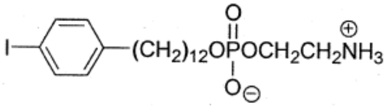

3

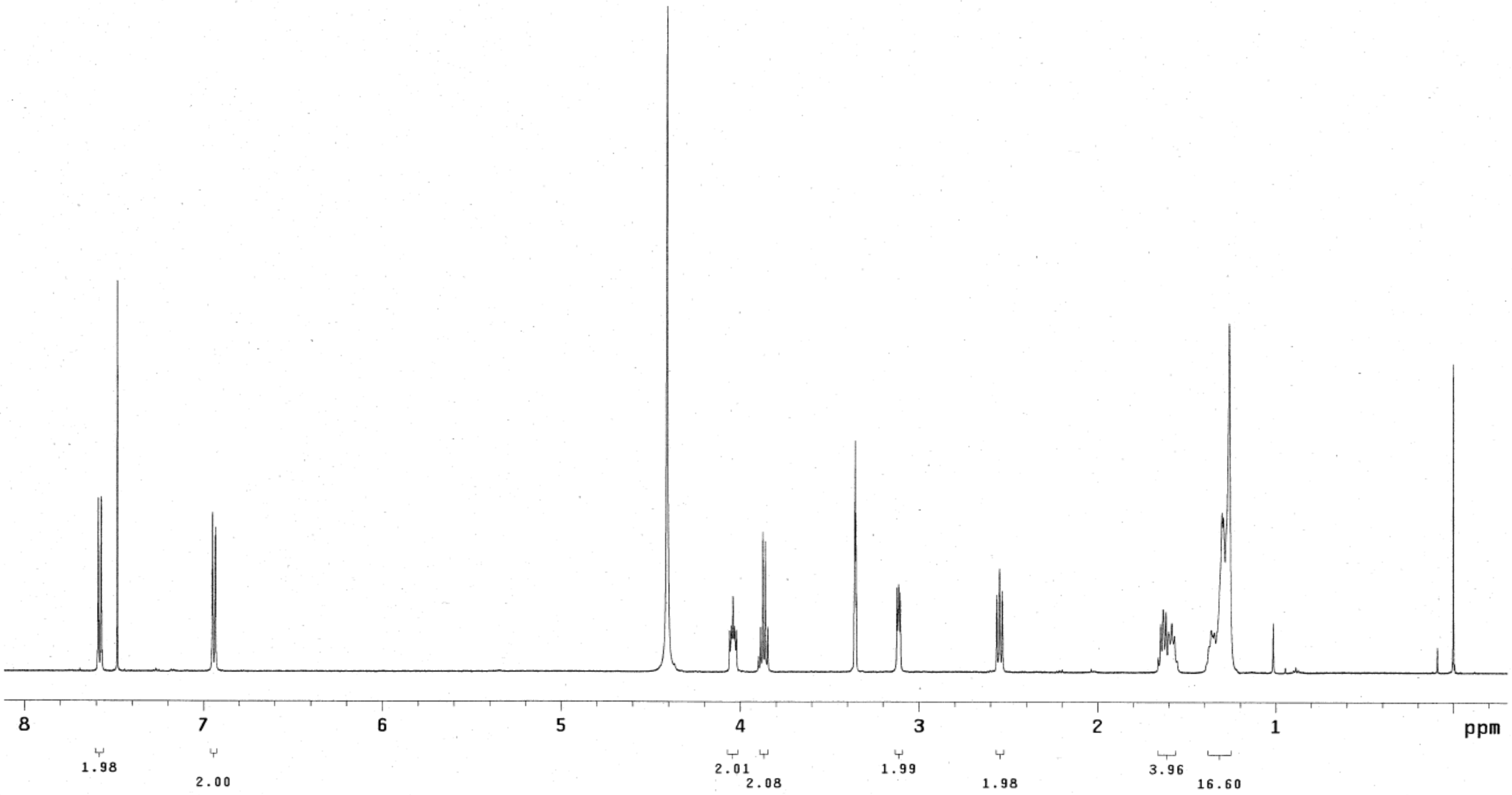

S5

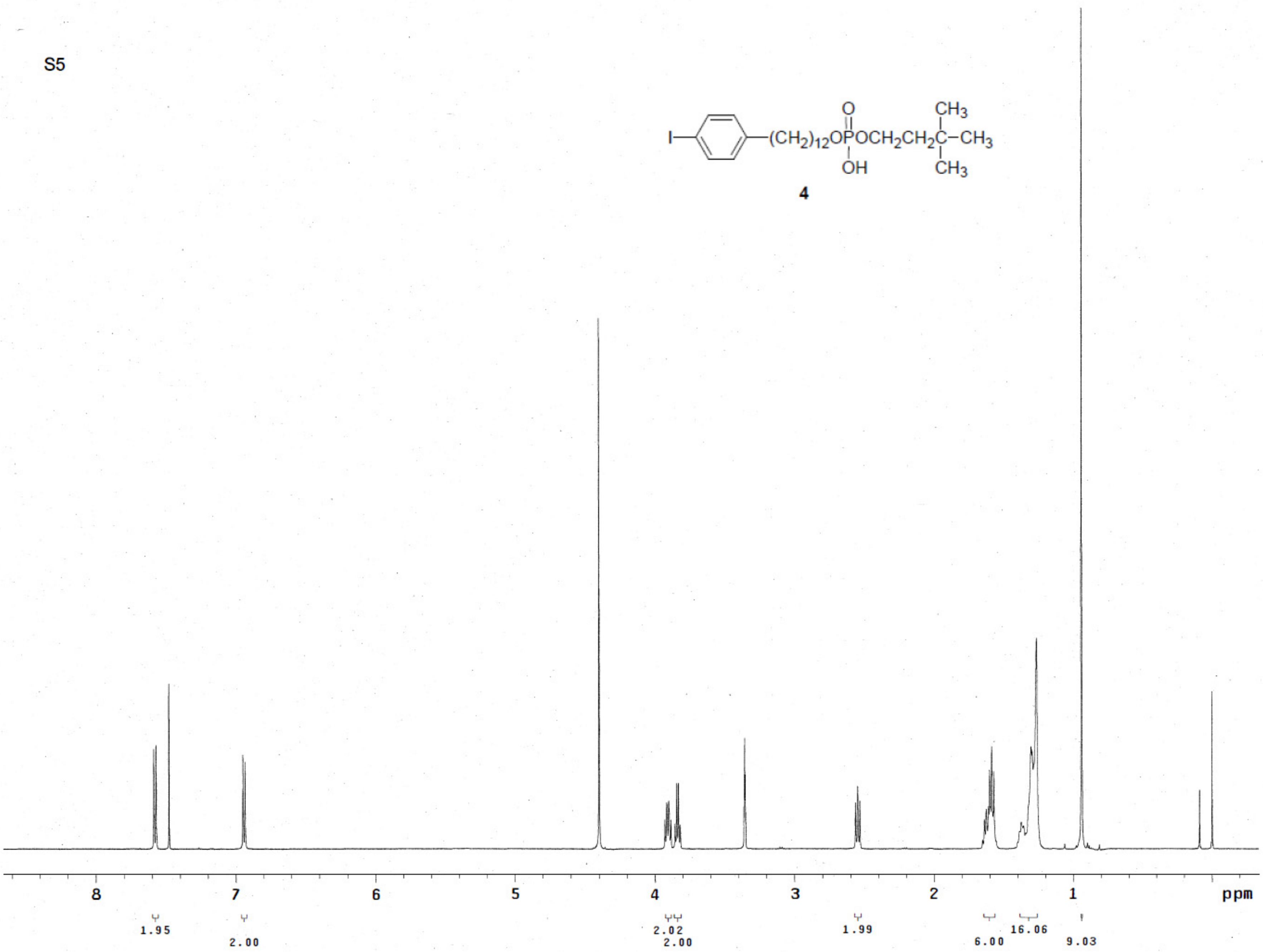

I—C6H4—(CH2)12OP(O)(OH)OCH2CH2C(CH3)3
4
8
7
6
5
4
3
2
1
ppm
1.95
2.00
2.02
2.00
1.99
6.00
16.06
9.03
